# Aspects Regarding the Consumption of Dietary Supplements among the Active Population in Romania

**DOI:** 10.3390/ijerph20010850

**Published:** 2023-01-02

**Authors:** Pia-Simona Fagaras, Silvia-Violeta Teodorescu, Anca Bacarea, Renato-Gabriel Petrea, Adela-Ioana Ursanu, Geanina Cozmei, Liliana-Elisabeta Radu, Gynetta-Ionela Vanvu

**Affiliations:** 1 Department of Doctoral Studies, National University of Physical Education and Sport Bucharest, 140 Constantin Noica Street, 060057 Bucharest, Romania; 2 Department of Human Movement Sciences, “George Emil Palade” University of Medicine, Pharmacy, Science, and Technology of Targu Mures, 38 Gheorghe Marinescu Street, 540139 Targu Mures, Romania; 3 Department ME1–Faculty of Medicine in English, “George Emil Palade” University of Medicine, Pharmacy, Science, and Technology of Targu Mures, 38 Gheorghe Marinescu Street, 540139 Targu Mures, Romania; 4 Faculty of Physical Education and Sport, “Alexandru Ioan Cuza” University of Iasi, 11 Carol I Avenue, 700506 Iasi, Romania; 5 Department of Teacher Training, “Gheorghe Asachi” Technical University of Iasi, 67, Professor Dimitrie Mangeron Avenue, 700050 Iasi, Romania; 6 Department of Preventive Medicine and Interdisciplinarity, “Grigore T. Popa” University of Medicine and Pharmacy, 16 Universității Street, 700115 Iasi, Romania

**Keywords:** dietary supplements, physical activity, lifestyle, health

## Abstract

Food supplements contain a variety of combinations of vitamins, minerals, plant extracts, and other substances. Any physical effort requires energy from balanced and healthy nutrition. This research aimed to identify the categories of dietary supplements used by active and very active participants who attend the sports and leisure centers from Targu Mures city. The survey was developed in paper and online forms using the Internet and supported multimedia and self-administration. The sample consists of 517 subjects chosen randomly from the research population and divided into the active and highly active PAI subgroups based on age, education, and gender. IBM SPSS Statistics Version 20.0 was used to process the collected data. All data were analysed descriptively. Differences between independent groups were assessed using an Independent sample t-test and a Chi-square test. Statistical significance was accepted when *p* < 0.05. Additionally, a Pearson correlation was done. The most representative age category was between 18 and 22 years old (42.7%), the ones using dietary supplements most frequently. There were significant differences by group of age regarding the consumption of food supplements (*p* < 0.05) and also by gender (*p* < 0.05)**.**

## 1. Introduction

The benefits of exercise for the population are often presented in the literature [1]. According to the World Health Organization (WHO), physical activity contributes to preventing and managing no infectious diseases such as cardiovascular diseases, cancer, and diabetes, reduces symptoms of depression and anxiety, and improves overall well-being. Insufficiently active people have a 20% to 30% increased risk of death compared to sufficiently active ones. The WHO defines physical activity as any bodily movement produced by skeletal muscles that require energy expenditure [2]. Regular physical activity associated with a balanced diet is an essential factor in our health [3].

The Physical Activity Index (PAI) is the most commonly used questionnaire in Romania to assess health-related physical fitness. It comprises three items based on the intensity of the effort, the time spent attending the physical activity, and the frequency of participation in PA [4,5,6]. The score of PAI is obtained by multiplying the given answer for items and the results show the level of physical activity, from sedentary (20 points) to a very active lifestyle, with an excellent level of PA (81–100 points) [7].

To increase the population’s PA level, sports and leisure centers are ideal for providing PA equipment within gyms, exercise class activities, and swimming pool facilities [8]. Gyms have been a place dedicated to improving physical exercise in a controlled environment and also offer various indoor activities [9]. Food and supplement consumption habits have also changed to maintain a fit lifestyle during everyday activities and professional careers. As a result, dietary supplements have increased among people of all ages [10,11,12].

Therefore, for a healthy life, the WHO makes a series of recommendations regarding the number of calories per day, limiting the consumption of sugar, salt, and saturated fat, increasing the consumption of vegetables and fruits, and a diet with essential micronutrients, such as vitamins and minerals [13].

According to European legislation, food supplements represent concentrated sources of nutrients or other substances with a nutritional or physiological effect, and whose purpose is to supplement the diet. Therefore, they are marketed in the form of capsules, lozenges, tablets, pills powder sachets, liquid ampoules, dropper bottles, and other similar liquids and powders intended to be taken in small measured unit quantities [14]. Scientists and health professionals agree that dietary supplements may benefit human health under certain conditions. Still, they should not replace the complete and balanced daily meals with foods necessary for a healthy diet [13,15,16].

Numerous pieces of evidence paid attention to nutritional supplements essential for sports performance and physical activity [17,18,19,20,21,22]. Dietary supplements contain a wide variety of ingredients including macro and micronutrients (e.g., minerals, vitamins, proteins, amino acids) and ergogenic supplements (e.g., creatine, caffeine, β-alanine), among others [23]. According to Maughan et al. (2018), nutritional supplements can be classified as dietary supplements, sport nutrition products, or ergogenic supplements. The category of dietary supplements mainly includes micronutrient supplements, such as vitamins and minerals, but also essential fatty acids. The main objective of supplementation is the compensation of nutrient deficiencies due to inadequate intake and/or increased need. Macronutrients, namely carbohydrates, proteins, and fats are contained in sport nutrition products, such as sports drinks, recovery drinks, energy bars, etc., while ergogenic supplements, including caffeine, beta-alanine, carbohydrates, sodium nitrate, and creatine, are products that are claimed to have performance-enhancing properties [21].

Globally, the market for dietary supplements is constantly growing. However, excessive consumption of these nutritional supplements can have detrimental consequences on the body [24]. Related to physically active subjects, in previous studies, the main reason for dietary supplementation were muscle building, weight loss, and health benefits [25,26,27,28].

Increasing interest in food supplements and increasingly abusive and uncontrolled consumption requires an increasingly rigorous attitude from the authorities and national nutrivigilance.

Various studies on dietary supplements used by the active population have been conducted internationally, but none of these were performed in Romania. The study aimed to investigate (1) the type of dietary supplement intake; (2) the sources of supplement acquisition; (3) the effect of dietary supplements used by the active population from Targu Mures city. Furthermore, information on the reason for consuming dietary supplements, and the type of supplements used according to gender, age, and PA level may provide insights into the need for age-specific education on the safe use of supplements for improved health, body composition, and physical fitness.

## 2. Materials and Methods

### 2.1. Sample Design

The study’s general research sample consisted of Targu-Mures City residents aged 18 to 62 who attended sports and leisure centers.

Respondents were selected for having a Physical Activity Index (PAI) over 61. There were two groups of physical activity in the layer: active (PAI between 61 and 80) and very active (between 81 and 100).

To determine the PAI [6,7] the participants were asked to indicate the level of participation in PA for three parameters (intensity, duration, and frequency) with four or five evaluation scales for scoring (Table 1). PAI is obtained by multiplying the score of each parameter and the interpretation of the result was made according to Table 2.

**Figure 1:**
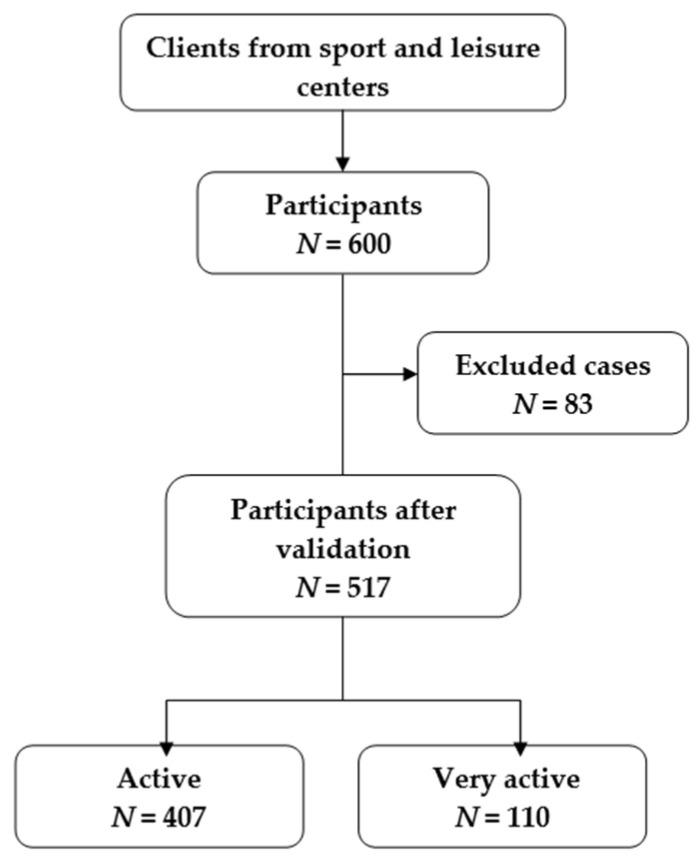
Characteristics of the participants.

Six hundred participants were recorded during the survey period and were observed as two PAI subgroups (Figure 1).

Participants were informed that their contribution was voluntary and anonymous and that they could withdraw without providing any reason. Eighty-three were excluded from processing the data due to incomplete data or not meeting the inclusion criteria.

The sample includes 517 subjects randomly drawn from the research population according to age, education level, gender, and PAI subgroups.

Table 3 displays the subjects’ distribution by age categories.

Regarding education (Table 4), out of the total number of respondents, 265 graduated high school (51.3%) and 252 graduated from a higher education institution (48.7%).

### 2.2. Instruments

The participants in this study had the choice to participate in the survey either on paper or online using Google Workspace’s web instrument form, which was built as part of the project [29] and allowed multimedia and self-administration [30]. The questionnaire was validated in a pilot study. During this process, 15 subjects completed the questionnaires in full and then were interviewed by a research member to obtain their feedback and suggestions for improvement. The 15 subjects who completed the pilot testing did not participate in the final survey. Moreover, the responses gathered during pilot testing were not included in the final analysis.

Cronbach’s Alpha was 0.786 which is greater than the acceptable range of 0.7 indicated score [31], and good according to Marôco (over 0.9: excellent, 0.8–0.9: very good, 0.7–0.8: good, 0.6–0.7: medium, 0.5–0.6: reasonable, below 0.5: bad) [32].

The purpose of the empirical study starts from the premise that active populations use dietary supplements to support physical effort in the gym or during performance training with expected effects.

In addition, the study’s overarching goal is to pinpoint the significant subcategories of dietary supplements consumed by the group’s elderly, active, and very active population. The objectives of the study consisted of:

- identifying the reasons why food supplements are consumed, and
- identifying how these food supplements are obtained.

Due to the lack of similar studies in the international and national literature and based on the authors’ professional experience in physical activity and supplement consumption. The research hypotheses of the study were intuitively established as follows:

**H1:**  *Young adults, residents in Targu Mures city who attend sports and leisure centers, between the 18–24 age group consume more food supplements than other age categories.*

**H2:**  *Active and very active men with over 61 Physical Activity Index score take more nutritional supplements than women.*

The questionnaire included personal data such as age, gender, weight, height, BMI, and level of education. Additionally, there are inquiries concerning the quality, quantity, and duration of physical activity.

If such supplements were taken, more inquiries were made regarding the kind of dietary supplements taken, how they were obtained, the sources of acquisition, and the anticipated effects.

### 2.3. Procedure

The survey is an extensive study designed to capture, in statistical form, the frequency of characteristics or characteristic variables (interests, attitudes, opinions, options) at the level of some populations [33].

Studies of beliefs, attitudes, motivation, and aspirations, in other words, studies of human life can become the center of complicated subject investigations. Nevertheless, it does not end with them [34].

The study was approved by the Ethics Committee of the University of Medicine and Pharmacy, Science, and Technology “G.E. Palade” Targu Mures and conducted under the Helsinki Declaration. The study also complied with all applicable laws about data protection, privacy rights, and personal information. All participants were made aware of the goal and aim of the study as well as the voluntary nature of their participation. They were free to leave the study without giving a reason.

The questionnaire was applied online by sending an invitation and later reminders through email and social media and printed to respondents attending sports and leisure centers. Respondents followed a link to the online survey, and informed consent was assumed from the respondents’ compilation. Printed forms were distributed in the sports and leisure centers.

We considered a questionnaire valid only when that questionnaire was completed in full by the respondents from Targu Mures city and considered active and very active according to the Physical Activity Index.

All data were recorded into Google Workspace automatically for the online survey and manually for the printed version.

### 2.4. Data Analysis

The Excel database was exported to Statistical Package for the Social Sciences (SPSS 20 IBM for Windows). It included descriptive statistics of mean, SD, and the frequency of occurrence expressed in absolute values or percentages. Additionally, an Independent sample t-test was performed for the following variables: age, height, and weight. Chi-square tests were applied to verify the distribution of answers regarding the variables used. A Pearson correlation (r) was applied to determine the correlations between variables in the “type of dietary supplements” category. A *p*-value under 0.05 was considered statistically significant. The item analysis was performed using Pearson correlation coefficients, and the associations were interpreted as not existing (r = 0), very weak (0.00 < r < 0.10), weak (0.10 ≤ r < 0.30), moderate (0.30 ≤ r < 0.50), strong (0.50 ≤ r < 0.70), very strong (0.70 ≤ r < 1), or perfect (r = 1), according to the value of r [35].

## 3. Results

### 3.1. Characteristics of the Study Population

The study population was comprised of 264 (51.06%) males and 253 (48.93%) females, categorized according to PAI as active and very active people. Most participants from the active population have a normal BMI (*N* = 370, 63.8%), while males were 136 (23.45%) and females were 194 (40.35%) (Table 5).

### 3.2. Consumption of Dietary Supplements

Of 517 study participants, 240 reported consuming dietary supplements. Table 6 displays the distribution of dietary supplement intake by age group.

There was a significant difference between the observed and expected frequencies in the case of age categories regarding the preference for the use of dietary supplements (x^2^ = 25.010. df = 8, *p* = 0.002).

Vitamins (68%) and proteins (64.1%) are the supplements that people in the population who assumed dietary consumption use the most, followed by amino acids (23.8%) and minerals (39%). The participants that consumed supplements stated the following sources of purchase: instructors (48.1%), pharmacies (24.7%), the Internet, TV (21.3%), profile store (17.4%), and colleagues (12.3%). Therefore, it was determined that gaining muscle mass (127, 54.5%), energy and endurance (129, 55.4%), and post-exercise recovery (113, 48.5%) were the primary motivations for using nutritional supplements (Table 7).

Making a correlation between the types of supplements (Table 8) we observe that there are statistically significant correlations between the types of food supplements.

## 4. Discussion

Our study aimed to investigate the consumption of dietary supplements and opinions about the type of supplements used, sources of purchase, and the effects among active and very active lifestyle populations. The results from this research are of particular interest because there is much information on the use of dietary supplements in the inactive population, and a lack of data on people who attend sports centers and leisure activities for a healthy lifestyle. Dietary supplement use is common among recreational and professional athletes [36,37]. The present study showed the consumption of dietary supplements among people (18–62 years). The prevalence of consumption of supplements was 46.42% among the population with a higher physical activity level. In our target group, we observed that consumption of supplements decreased by age group; the main category consuming dietary supplements is 18–22 years, and significant differences were found between age groups regarding consumption. According to previous research, supplement use rises with age; women are more likely than men to use supplements, and people who routinely exercise to maintain a healthy lifestyle report the lowest prevalence of supplement use [37,38,39]. In addition, a healthy lifestyle implies a healthy diet and it is understood that followers of this lifestyle will not seek to use dietary supplements [40,41,42].

The most popular supplements consumed by the study population were vitamins, followed by proteins, minerals, and amino acids. We discovered a medium to strong correlation (r = 0.615) between vitamins and minerals (who consumes vitamins, also consumes minerals); a medium correlation (r = 0.437) between minerals and amino acids (who consumes minerals also consumes amino acids); a poor correlation (r = 0.357) between amino acids and vitamins (not everyone who consumes vitamins also consumes amino acids); poor to average correlation (r = 0.340) between protein and vitamins (not everyone who consumes vitamins also consumes protein). Some studies have shown that the most popular dietary supplements among amateur athletes are usually sports energy drinks, vitamins and minerals, caffeine, creatine, and protein supplements [43,44,45,46,47,48]. In an Australian survey, multivitamins/multiminerals and fish oils were the most frequently reported supplements [49].

Today, dietary supplements are widely used in modern society. Marketing claims for some dietary substances include improvements in overall health status, enhancement of cognitive or physical performance, an increase in energy, the loss of excess weight, attenuation of pain, and other favorable effects [43]. Our results showed that males consume more proteins and ergogenic supplements associated with maintaining and improving muscle mass and strength, which increases muscle energy, endurance, and lean muscle mass, than females. Similar results were obtained in other studies [43,44,45,46,47,48].

People frequently take supplements without thinking about possible side effects, mainly because they are said to be substances that improve fitness. However, over time, overconsumption of dietary supplements can alter health and performance [50].

Gender-based differences in sports and exercise nutrition have been considered an important area of debate, particularly during the recent two decades [44,45,46,47]. In our study male subjects consume more dietary supplements than female subjects. Similar results were found in other studies [51].

We discovered that 48.1% of the instructors recommended supplements which is similar to the findings of Finamore et al. (2022) with 27.9% [52]. Overall, instructors, pharmacy (24.7%), the internet, and television information are the top three sources for purchasing supplements. People from the entourage (family, friends, teammates), coaches and/or instructors, as well as supplement seller websites, are typically those who recommend the use of food supplements, to the detriment of qualified people (dietitians, nutritionists, sports doctors) [15,28,53,54,55].

The present sample was characterized by using vitamins and proteins to increase muscle mass, obtain energy and endurance, and recover post-exercise at instructors’ suggestion from sport leisure centers. Moreover, consumers of dietary supplements are young males.

## 5. Conclusions

This study provides original information on dietary supplement use, type, effects, and source for purchasing among healthy and active lifestyle residents in the city of Targu Mures, Romania. DS were used by 240 participants (50.29%). Vitamins are the most frequently consumed supplement followed by proteins. Results also indicate that both genders use supplements for energy and endurance, increased muscle mass, and post-exercise recovery, and instructors represented a reference source for prescription supplements. Our findings can raise awareness about the importance of the acquisition, clean and healthy consumption of dietary supplements, and the need to consult a nutrition specialist before using these products. Nutritional supplements should not be encouraged to the detriment of adequate nutrition.

## Figures and Tables

**Table 1:** Level of participation in physical activity.

Parameters	Scoring	Characteristics
	5	The effort that leads to rapid breathing and sweating
	4	Efforts increasing the respiratory rate and sweating
Intensity	3	Effort above average
	2	Moderate effort
	1	Easy effort
	4	Over 30 min
Duration	3	20–30 min
	2	1–20 min
	1	Under 10 min
	5	Daily or almost daily
	4	3–5 times weekly
Frequency	3	1–2 times weekly
	2	Several times monthly
	1	Less than once monthly

**Table 2:** Physical activity index.

Score	Characterization	Physical Activity Category
81–100	Very active lifestyle	Superior
61–80	Active lifestyle and healthy	Very good
41–60	Acceptable	Reasonable
21–40	Insufficient activity, relatively sedentary	Weak
≤20	Sedentary	Very weak

**Table 3:** Subjects’ distribution by age categories.

Age Category (Years)	Frequency	Percent
18–22	221	42.7
23–27	86	16.6
28–32	71	13.7
33–37	41	7.9
38–42	36	7.0
43–47	27	5.2
48–52	18	3.5
53–57	14	2.7
58–62	3	0.6
Total	517	100.0

**Table 4:** Distribution of subjects according to the last school graduated.

School Graduated	Frequency	Percent
High school	265	51.3
Undergraduate	142	27.5
Master degree	93	18.0
PhD	10	1.9
Postgraduate	7	1.4
Total	517	100.0

**Table 5:** Characteristics of the study population.

Variables	Total		Male		Female		*p*
Age	28.04 ± 10.29		27.03 ± 10.03		29.09 ± 10.47		0.024 ^a^
Height	174.02 ± 9.56		180.09 ± 9.51		167.34 ± 6.93		0.000 ^a^
Weight	72.50 ± 15.17		82.31 ± 12.08		62.24 ± 10.60		0.000 ^a^
BMI							
<18.5	20 (3.97%)		2 (0.39%)		18 (3.58%)		
18.5–25.0	370 (63.8%)		136 (23.45%)		194 (40.35%)		
25.1–30.0	139 (36,9%)		105 (20.32%)		34 (6.58%)		
30.1–35.0	26 (5.0%)		19 (3.65%)		7 (1.85%)		
35.1–40.0	1 (0.2%)		0 (0%)		1 (0.2%)		
>40.0	1 (0.2%)		1 (0.2%)		0 (0%)		
							0.000 ^b^
PAI							
Very active lifestyle	110 (21.3%)		72 (13.9%)		38 (7.4%)		
Active lifestyle	407 (78.7%)		186 (36.0%)		221 (42.7%)		
							0.001 ^b^

^a^ Independent sample t-test; ^b^ Chi-Square.

**Table 6:** Consumption of dietary supplements by age categories.

Age Category (Years)	Total	Percent
18–22	87	36.25
23–27	54	22.50
28–32	41	17.08
33–37	18	7.50
38–42	11	4.58
43–47	11	4.58
48–52	8	3.33
53–57	7	2.92
58–62	3	1.25
Total	240	100

**Table 7:** Multiple response frequency.

Items	Total	Male	Female
Type of the supplement intake			
Vitamins	157 (68%)	85 (36.8%)	72 (31.2%)
Minerals	90 (39%)	54 (23.4%)	36 (15.6%)
Amino acids	55 (23.8%)	38 (16.5%)	17 (7.4%)
Proteins	148 (64.1%)	85 (36.8%)	66 (27.3%)
Source of purchasing supplements			
Pharmacy	58 (24.7%)	19 (8.1%)	39 (16.6%)
Profile store	41 (17.4%)	22 (9.4%)	19 (8.1%)
Colleagues	29 (12.3%)	23 (9.8%)	6 (2.6%)
Internet, TV	50 (21.3%)	28 (11.9%)	22 (9.4%)
Instructors	113 (48.1%)	59 (25.1%)	54 (23.0%)
The effect of dietary supplements			
Increase muscle mass	127 (54.5%)	81 (34.8%)	46 (19.7%)
Weight loss	33 (14.2%)	13 (5.6%)	20 (8.6%)
Energy and endurance Post-exercise recovery	129 (55.4%) 113 (48.5%)	74 (31.8%) 72 (30.99%)	55 (23.6%) 41 (17.6%)
Chondroprotective	44 (18.9%)	24 (10.3%)	20 (8.6%)

**Table 8:** Correlation between types of supplements.

	TSU_Vitamins	TSU_Minerals	TSU_Amino-Acids	TSU_Proteins
TSU vitamins	1	0.615 **	0.357 **	0.340 **
TSU minerals		1	0.437 **	0.232 **
TSU amino acids			1	0.399 **
TSU proteins				

TSU = Type of supplements used; ** Correlation is significant at the 0.01 level (2-tailed).

## Data Availability

Not applicable.
